# Harnessing Technology to Foster Social Connection and Enhance Well-Being Among Older Adults

**DOI:** 10.1093/geroni/igaf122.1328

**Published:** 2025-12-31

**Authors:** Walter Boot, Edie Sanders

## Abstract

Existing and emerging technologies offer powerful tools to foster social connection and enhance well-being among older adults. From digital communication platforms to personalized digital health tools, these innovations can help reduce loneliness, support mental health, and promote meaningful engagement. As the older population grows and diversifies, leveraging technology to meet their social and emotional needs has become both an opportunity and a necessity. This symposium brings together researchers advancing our understanding of how technology can support connection and quality of life in later life. Dr. Kalon Sou will present findings from a study evaluating older adults’ experiences with an age-friendly, AI-powered chatbot, identifying key factors—such as empathy, sociability, and agency—that shape willingness to use social chatbots. Nicole Memmer will explore how different types of Internet use, especially for social purposes, relate to older adults’ offline participation, with digital skills and local context influencing the benefits gained. Dr. Paul P. Freddolino will highlight a multi-year initiative to increase digital health literacy among hard-to-reach older adults using community-based strategies, emphasizing the role of trust and early outcomes related to technology engagement. Dr. Ines Simbrig will examine how aging-in-place technologies affect older adults’ perceptions of age-related change, revealing a complex trade-off between feelings of safety and potentially negative shifts in self-perception. Finally, Dr. Yong Kyung Choi will introduce the Adult Wellbeing CheckUp, a web-based platform that offers multi-domain assessments and personalized recommendations to help older adults and care partners improve health, engagement, and quality of life.

